# Intravenous Immunoglobulin Therapy in Livedoid Vasculopathy: Retrospective Observation of Clinical Outcome and Patient’s Activity Level

**DOI:** 10.1177/12034754211003525

**Published:** 2021-03-28

**Authors:** Katrin Kofler, Anke Strölin, Vanessa Geiger, Lukas Kofler

**Affiliations:** 1 9188 Department of Dermatology, Eberhard-Karls University, Tübingen, Germany

**Keywords:** livedoid vasculopathy, intravenous immunoglobulin, ulceration, livedo reticularis

## Abstract

**Background:**

Livedoid vasculopathy (LV) is a rare disease characterized by livedo racemosa, atrophie blanche, ulcerations, and severe pain. Low molecular weight heparins and rivaroxaban can be used in LV-patients. In addition, intravenous immunoglobulins (IVIG) have been described as treatment-option.

**Objectives:**

Objective was to investigate the therapeutic effect of IVIG on ulcer, pain and restrictions in daily life.

**Methods:**

Thirty-two LV-patients who received IVIG at the Department of Dermatology Tübingen between 01/2014 and 06/2019 were identified. Twenty-five of these patients were available for further follow up and were included in the study. Patients were interviewed using a questionnaire focusing on the course of the disease, symptoms, and subjective response to IVIG-treatment.

**Results:**

Twenty-five patients were included in the study (mean follow up: 28.9 months). Patients received an average of 6.8 cycles (range 1-45) of IVIG during the observed period.

Significant improvements were seen regarding skin findings, pain, and limitation of daily activities. Complete remission of symptoms was observed in 68% of patients. Good tolerability of IVIG was shown in 92%.

**Conclusions:**

A good therapy response regarding ulceration, pain, and daily life restrictions with good tolerability was demonstrated for IVIG (2 g/kg bodyweight over 5 days).

## Introduction

**Figure 1:**
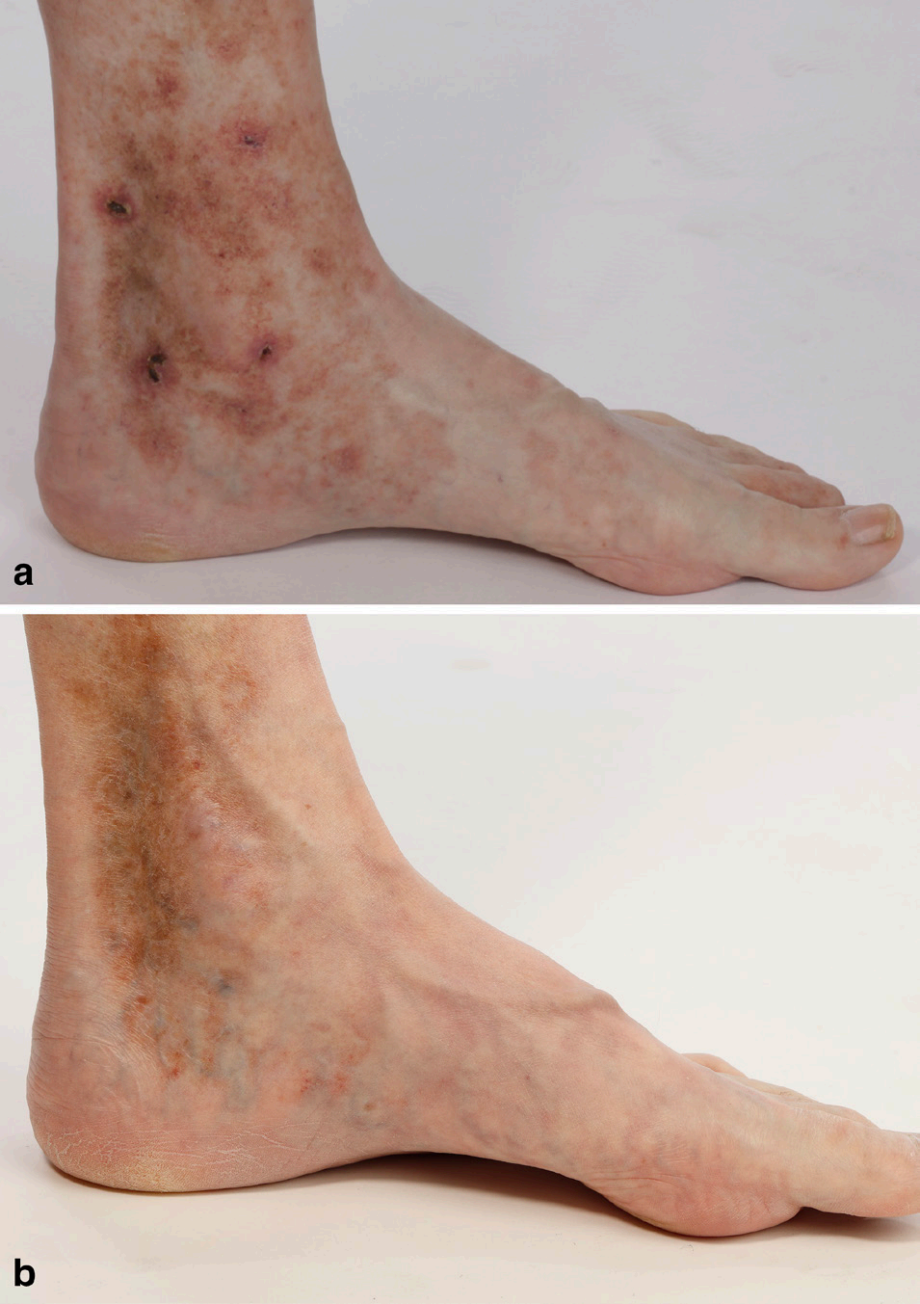
(a) Prominent bizarrely configured ulcerations with perifocal erythematous margins at the inner ankle of a patient prior to therapy with IVIG (view from medial). Clearly visible are atrophic blanche and postinflammatory hyperpigmentations. (**b**) Skin findings on the patient’s inner ankle under ongoing IVIG therapy, taken 10 months after the start of the therapy (view from medial). Complete healing of ulcerations and erythema; only pale postinflammatory hyperpigmentations are visible. IVIG, intravenous immunoglobulins

Livedoid vasculopathy (LV) is a rare disease with recurrent thrombotic occlusion of cutaneous vessels of the lower extremity. The causative pathomechanism, which leads to formation of fibrin thrombi and the disturbance of the microcirculation, is not yet fully understood. Procoagulant mechanisms have been described but are not detectable in all affected patients.^1-3^ The typical clinical triad consists of livedo racemosa, ulceration and atrophie blanche (Figure 1). Women are more often affected than men; in a recent study a gender ratio of 2.1:1 has been reported.^4,5^

It has been shown, that the symptoms of the disease and its consequences considerably reduce the quality of life.^6^ As patients experience severe pain, especially due to local ischemia, extensive ulceration and rapid irreversible scarring, quick and efficient treatment options are essential. However, no approved drug-based treatment is currently available for LV. Most experience in Germany is available for low molecular weight heparins.^4,7,8^ Recent study results also showed effective pain reduction using rivaroxaban.^9^ Antithrombotic therapy is intended to prevent or positively influence microcirculatory thrombotic events. Although pain reduction and symptom relief can be achieved in many patients with these therapies, not all of them show a sufficient therapeutic response. Therefore, strategies for therapy-refractory LV are warranted. Both, case reports and studies with smaller case numbers indicate a good response for intravenous immunoglobulins (IVIG) in the treatment of LV.^10-13^ In addition to the anti-inflammatory and immunomodulatory effect of IVIG, anticoagulant effects via modulation of endothelial function, inhibition of thrombogenic antibodies such as antiphospholipid antibodies and reduction on platelet adhesion has been postulated.^14-17^

Objective of this study was to investigate patients’ satisfaction and subjective experience of the therapeutic effect of IVIG in a dose of 2 g/kg bodyweight administered over a period of 5 days every 4 weeks regarding ulcer healing, pain symptoms and restrictions in daily life.

## Material and Methods

Patients were identified through a search for the ICD code L95.0 in the documentation software used in our hospital (ISH, SAP, Walldorf/Germany). Subsequently, all patients who were coded with L95.0 were individually reviewed to to verify the diagnosis of LV.

A total of 32 LV-patients who received IVIG at a dose of 2 g/kg body weight every 4 weeks (25, 28 days) at the Department of Dermatology / University Hospital Tübingen between 01/2014 and 06/2019 were identified. Twenty-five of these patients gave their informed consent to participate in the current study. Data regarding further follow-up examinations were available from all 25 patients. Patients were interviewed once using a standardized questionnaire focusing on the course of the disease, symptoms and subjective response to IVIG-treatment and restriction in their daily life. In addition, regular follow-up examinations were performed, including clinical examination of the patients and laboratory analysis (differential blood count and renal function parameters).

The diagnosis of LV was made by board-certified dermatologists based on the following criteria: typical clinic with recurrent ulcers, livedo racemosa and atrophie blanche, supplemented by histopathological examination if indicated. The diagnosis was made for each individual patient after exclusion of possible differential diagnoses and careful consideration of all existing findings. Histological criteria for LV were fibrin thrombi and fibrin deposits in the vessel walls without significant vasculitis, possibly with evidence of erythrocyte extravasation.

The course of the disease was assessed in all included patients on the basis of inpatient and outpatient medical records. Information on the individual course of the disease was also obtained from the questionnaire. An ulceration was defined as a tissue defect of the skin that extends beyond the level of the epithelium. Erosion was defined as a localized superficial epithelial defect. Information about the skin findings, especially the presence of ulceration or erosion, was obtained from medical records. Every initiation of IVIG therapy was individually indicated by board-certified dermatologists on the basis of inadequate response to prior therapy and particularly rapid disease progression.

IVIG was administered intravenously at a dose of 2 g/kg body weight over a period of 5 days. A treatment cycle with IVIG was performed every 25 to 28 days. The total dose was split over 5 days to reduce the daily intake of IVIG and to reduce renal exposure. IVIG was administered in an in-patient setting. In addition to IVIG administration, in-patient treatment allows rapid and sufficient adjustment of pain therapy as well as short-term wound controls.

The current study was approved by the Ethics Commission of the University of Tuebingen (Number 004/2019BO2). All patients had given their informed consent.

All collected data were analyzed using JMP (SAS Institute Cary/NC, USA). Clinical and demographic characteristics were evaluated statistically.

Numerical variables were described by mean value. Pearson’s Chi-Square-Test and Fisher’s exact test were used for the analysis. *P* values < .05 were considered statistically significant.

## Results

### Study Cohort

A total of 25 patients were included in the study (60.0% female, 40.0% male) with a mean follow up time of 28.9 months. Patients received an average of 6.8 cycles (range 1-45) of IVIG during the observed period. The average patients’ age at the time of the first IVIG cycle was 66.4 years (range 46-83).

Most patients showed LV on the lower leg (52%), ankle (32%), and foot (16%). The most frequent secondary diagnoses were arterial hypertension (72%), diabetes mellitus (24%), thrombosis (24%), history of a malignant tumor (24%), peripheral arterial occlusive disease (16%), and rheumatoid arthritis (16%) (Table 1).

**Table 1:** Patients Characteristics.

Age (mean), years	66.4
Sex	
Male	15
Female	10
Localisation of initial manifestation	
Lower leg	13
Ankle	8
Foot	4
Pretherapy before for IVIG	
Cortison	15
Acetylsalicylic acid	12
Direct anticoagulation/vitamin K-antagonists	10
Heparin	5
Other immunosuppressive drugs	2
Prostaglandins	1
None	2
Improvement under pretherapy	
Yes	2
No	21
Comorbidities	
Hypertension	18
Diabetes mellitus	6
Peripheral arterial disease	4
Thrombosis	6
History of tumor	6
Rheumatoid arthritis	4
Systemic lupus erythematosus	2
Löfgren syndrome	1
Thrombophilia	
Antiphospholipid antibodies	4
Heterozygous prothrombin mutation	1
Heterozygous Factor V Leiden	1
Antithrombin deficiency	1

Abbreviation: IVIG, Intravenous Immunoglobulins.

The majority of patients (72%) received the diagnosis of LV within 12 months after onset of symptoms. However, in 16% of patients, it took more than 10 years until the final diagnosis of LV was made.

Forty percent of patients (10/25) reported symptoms such as ulcers and pain up to 6 months before initiation of IVIG therapy, while 28% (7/25) of patients experienced symptoms between 6 and 12 months before IVIG was initiated. 20% (5/25) of the patients reported that symptoms were present more than 10 years before IVIG treatment.

A total 16 patients (64%) were tested for antinuclear antibodies (ANA), which were detected in 6 of them (37.5%) with titers from 1:320 to 1:2560. 16 patients were screened for anti-neutrophil cytoplasmic antibodies (pANCA/cANCA), with all patients tested showing negative results.

In 28% of the patients (7/25), a coagulation disorder with varying clinical relevance was recorded, with antiphospholipid antibodies being found in 4 patients. A heterozygous factor V Leiden-mutation, a prothrombin mutation, and an antithrombin deficiency was recorded in one patient each (Table 1).

Before IVIG was administered, a large proportion of patients received other treatments (23/25; multiple options were possible). The most common pre-treatment regimens were systemic corticosteroid (60%) and acetylsalicylic acid (48%). 40% of patients received direct anticoagulants (rivaroxaban, apixaban) or vitamin K-antagonists, another 20% received low molecular weight heparin intracutaneously (Table 1).

Co-medication (aspirin, heparin, oral anticoagulants) was administered in 92% of patients during IVIG-treatment. In 57% of these patients, the co-medication was initiated due to pre-existing cardiovascular conditions (atrial fibrillation, coronary heart disease, thrombosis). The other patients received heparin or oral anticoagulants as first-line therapy for LV. Analgesic therapy was given individually and adjusted according to WHO guidelines if necessary. Compression therapy was performed after exclusion of contraindications (such as peripheral arterial disease).

### Treatment Response

**Figure 2:**
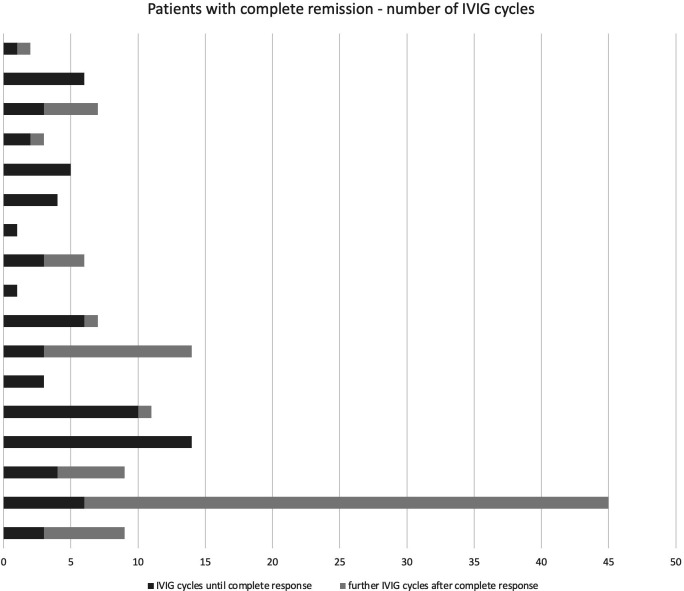
Patients with complete remission to IVIG therapy. the number of IVIG cycles until complete remission of symptoms was achieved is displayed in dark gray. Any further cycles are displayed in light gray. IVIG, intravenous immunoglobulins.

An improvement in clinical symptoms (pain, ulceration) was observed in 96% of patients (24/25) under therapy with IVIG. The improvement was observed within the first 6 months of treatment in 88% of patients (22/25). A complete remission of all symptoms was observed in 68% of patients (17/25) after a mean of 4.4 cycles of IVIG (range 1-14; Figure 2). Eleven patients had therapy discontinued immediately after complete remission or after the following cycle, while 6 patients received further IVIG therapy after complete remission. Two patients showed complete remission but again symptoms within less than 3 months, so therapy was continued. One of these patients still remains on IVIG therapy and received 45 cycles within the study period.

**Figure 3:**
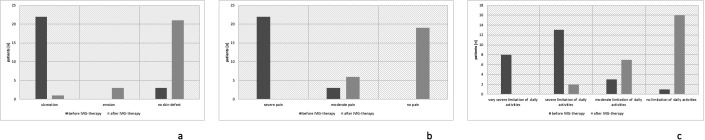
(**a**) Skin changes over the course of IVIG therapy classified as ‘ulceration’, ‘erosion’ or ‘without skin defect’ (dark grey = before IVIG therapy, light grey = after IVIG therapy) The Y-axis shows the absolute number of patients. (**b**) Pain sensation over the course of IVIG therapy classified as ‘severe pain’, ‘moderate pain’ or ‘no pain’ (dark grey = before IVIG therapy, light grey = after IVIG therapy) The Y-axis shows the absolute number of patients. (c) Representation of the impairment of daily life by the disease over the course of IVIG therapy classified as ‘very severe limitation of daily activities, ‘severe limitation of daily activities’, ‘moderate limitation of daily activities’ or ‘no limitation of daily activities’ (dark grey = before IVIG therapy, light grey = after IVIG therapy). The Y-axis shows the absolute number of patients. IVIG, intravenous immunoglobulins.

A significant improvement was observed regarding skin findings after IVIG therapy (*P* < .001). Ulcerations were recorded in 88% (22/25) of patients before IVIG-treatment. After treatment 84% (21/25) of patients displayed no more skin findings while one patient showed ulcerations and 3 patients showed erosions (Figure 3(a). We also observed a significant improvement of pain under therapy with IVIG (*P* < .001). Prior to IVIG-treatment, patients reported severe pain in 88% (22/25) and moderate pain in 12% (3/25). After IVIG-treatment, the majority of patients (76%; 19/25) reported to be free of pain, while 24% (6/25) of patients reported moderate pain and no more patient indicated severe pain during the follow-up period (Figure 3(b). 84% (21/25) of patients reported to experience a very severe or severe limitation of their daily activities by LV (Figure 3(c). These restrictions were shown to be significantly reduced by IVIG therapy (*P* < .001) .

Overall, good tolerability of IVIG in our patient cohort was shown in 92% of cases (23/25). One patient experienced an episode of headache, nausea and dizziness under therapy. In another patient dizziness, nausea and circulatory problems were reported under therapy.

## Discussion

Although LV is a rare disease, it poses a great challenge in clinical practice regarding diagnosis and therapy. An approved drug-based therapy is not available at present. It has been demonstrated that patients with LV benefit from low-molecular-weight heparins or rivaroxaban.^4,7-9^ In addition, a response to IVIG was shown in smaller collectives.^10,12,18^

LV is often associated with extreme pain and severe restrictions in patients’ private and professional lives. This study shows a significant improvement in the symptoms of LV in terms of pain, ulceration and restrictions in everyday life under therapy with IVIG. A treatment with IVIG 2 g/kg bodyweight for 5 days every 4 weeks led to an improvement of typical symptoms in 96% of patients, a complete healing of the symptoms could be achieved in 68% of patients. This was achieved after a mean of 4.4 cycles of IVIG. In-patient administration of IVIG allows short-term changes in pain therapy as well as wound management and is indicated, for example, in cases of severe pain. However, out-patient administration of IVIG is possible and safe in specialized centers.

In total, a complete remission of ulcerations was observed in 84% of patients and complete relief from pain in 76% of patients. The previously reported restrictions in everyday life also showed a significant improvement as a result of IVIG-treatment, as about two thirds of the patients no longer reported restrictions in everyday life and less than one third experienced only minor restrictions. Therapy with IVIG was reevaluated individually before each cycle. A complete remission of all symptoms was observed in 17 patients. In 11 patients, therapy was discontinued immediately after complete remission or after the following cycle. In 6 patients, a continuation of therapy was individually indicated although complete remission was achieved. Because of the high costs of therapy, a continuous evaluation of the continuation of therapy is necessary. In particular, therapy should be paused in case of complete remission or stable findings for at least 6 months.

Monshi et al. reported that 59% of disease episodes improved after 3 cycles and 86% after 6 cycles when treated with IVIG 2 g/kg bodyweight for 2 to 3 days.^10^ The authors showed an improvement of pain, ulceration and DLQI in 11 patients.^10^ Further retrospective case series such as Bounfour et al. including 5 patients and Ozden et al. including 9 patients with IVIG 2 g/kg bodyweight over a period of 3 days also reported good therapeutic response regarding pain and ulceration.^13,18^

A multifactorial etiology has been discussed for LV. We observed pre-existing conditions in the majority of patients, with arterial hypertension being the most frequent (72%). This significantly increased prevalence in relation to the normal population is congruent with the data of Weishaupt et al., who observed arterial hypertension in 70% of patients.^4^ Thrombophilia was detected in 28% of the patients in our cohort. Comparable results were published by Hairston et al..^1^ Di Giacomo et al. reported procoagulatory findings in laboratory tests in 52% of LV patients.^2^ However, not all patients in this study were examined for coagulation disorders as part of clinical routine.

The majority of the patients received a comedication with antiplatelet aggregation inhibitors or oral anticoagulants additional to IVIG-treatment, mostly due to cardiovascular conditions such as coronary heart disease or atrial fibrillation. Therefore, a synergistic effect of anticoagulants and IVIG-treatment cannot be ruled out.

Adverse effects are rarely observed in IVIG-treatment and usually mild. However, headache, hypertension, flush, fever, nausea, vomiting, or dizziness were reported in the literature.^19^ Observational studies and case reports reported an increased risk of thromboembolic events associated with IVIG therapy.^20-22^ These data resulted in a boxed warning from the FDA in 2013. Kapoor et al. reported a low proportion of thromboembolic events in a recent analysis of neurologic patients with IVIG therapy, but also discuss underestimation of such events due to underreporting.^23^In a systematic meta-analysis by Ammann et al, no increased risk of thromboembolic events was found in more than 4000 patients with IVIG.^24^ However, limiting factors were the median age of the investigated patients of 47 years and the underrepresentation of patients with inflammatory as well as non-neurological diseases.^24^ Therefore, these results cannot be generalized and further risk factors for a thromboembolic event have to be considered. Additional anticoagulation could possibly reduce the risk of thromboembolic events with IVIG.

IVIG treatment was generally well tolerated in our patients. The total dose of IVIG (2 g/kg bodyweight) was applied over a period of 5 days in all patients in this study. In our experience, administration over 5 days results in fewer side effects and, in particular, shows more stable renal retention parameters. This regimen has been used in our clinic for many years. Daily monitoring of renal values and sufficient fluid intake must be ensured. We observed mild side effects in only 2 of 25 patients. Very rarely described severe side effects such as aseptic meningitis, anaphylactic reactions or renal failure were not observed in our group.^19^

The treatment with IVIG is associated with high costs.^25^ Therefore, IVIG treatment should be considered only in patients who have no or insufficient symptom reduction despite previous therapies with rivaroxaban or low-molecular-weight heparins.^4,7-9^ In Germany, IVIG is not an approved therapy for LV, but can be used as an off-label treatment after prior approval by the patient’s health insurance company.

This study was designed as a retrospective observational study and therefore, several limitations must be consaidered. First of all, the retrospective study design has to be mentioned. A considerable limitation of the present study is also the interviewed-based design. However, this study design was chosen as the patient’s personal experience is of great relevance in the context of disease-related stigmatization, individual satisfaction with the treatment and assessment of patient’s quality of life. Most patients received a comedication; the number of patients was too small to calculate subgroups for all comedications. Due to the long course of the disease and externally initiated pre-therapies, it is not possible to provide an exact chronological list of pre-therapies. Therefore, only the proportion of patients with co-medication during ongoing IVIG therapy was reported. Due to the retrospective character of the study design, it is not possible to completely rule out self-limitation of the disease.

## Conclusion

To the best of our knowledge, this study includes the largest number of LV patients treated with IVIG to date. At a dose of 2 g/kg bodyweight over 5 days, a good therapy response regarding ulceration, pain and restrictions in daily life with good tolerability was demonstrated for IVIG. Based on the available data, IVIG-treatment for LV should be considered if previous therapies failed to result in sufficient improvement of symptoms.

## Supplemental Material

Supplementary Material 1 - Supplemental material for Intravenous Immunoglobulin Therapy in Livedoid Vasculopathy: Retrospective Observation of Clinical Outcome and Patient’s Activity Level Click here for additional data file. Supplemental material, Supplementary Material 1, for Intravenous Immunoglobulin Therapy in Livedoid Vasculopathy: Retrospective Observation of Clinical Outcome and Patient’s Activity Level by Katrin Kofler, Anke Strölin, Vanessa Geiger and Lukas Kofler in Journal of Cutaneous Medicine and Surgery
