# Supplementary material for: Intravenous Immunoglobulin Therapy in Livedoid Vasculopathy: Retrospective Observation of Clinical Outcome and Patient’s Activity Level
Source: J Cutan Med Surg. 2021 Mar 28;25(5):504–10. doi: 10.1177/12034754211003525 (PMC8474298; doi:10.1177/12034754211003525)
Supplement: Supplementary Material 1 - Supplemental material for Intravenous Immunoglobulin Therapy in Livedoid Vasculopathy: Retrospective Observation of Clinical Outcome and Patient’s Activity Level [file sj-pdf-1-cms-10.1177_12034754211003525.pdf]

Questionnaire (translated from the German version)

part I

How much time before treatment with IVIG infusions at our clinic did symptoms of livedovasculopathy (disease) such as pain, ulcers (discontinuity of the skin) or related occur for the first time?

- ☐ 0 to 6 months
- ☐ 6 months to 1 year
- ☐ 1 year to 2 years
- ☐ 2 years to 4 years
- ☐ 4 years to 6 years
- ☐ 6 years to 8 years
- ☐ 8 years to 10 years
- ☐ over 10 years

How much time passed between the onset of the first symptoms of livedovasculopathy disease and the diagnosis?

- ☐ 0 to 6 months
- ☐ 6 months to 1 year
- ☐ 1 year to 2 years
- ☐ 2 years to 4 years
- ☐ 4 years to 6 years
- ☐ 6 years to 8 years
- ☐ 8 years to 10 years
- ☐ over 10 years

In which parts of your body did you experience symptoms of livedovasculopathy disease? (Multiple answers possible)

- ☐ lower leg
- ☐ thigh
- ☐ ankle
- ☐ back of the foot

other body parts:

____

Where did symptoms of the disease first appear?
(only single answer possible)

- ☐ lower leg
- ☐ thigh
- ☐ ankle
- ☐ back of the foot
- other body parts:

____

Has there been a worsening of symptoms under certain conditions?

- ☐ yes
  - ☐ in summer
  - ☐ in winter
  - ☐ when standing for a long time
  - ☐ with stress
  - ☐ other:________________________________
- ☐ no

Have you had any other drug therapy for your disease prior to treatment with IVIG infusions at our clinic?

- ☐ yes
  - ☐ Aspirin
  - ☐ Cortisone
  - ☐ Tablets for blood thinning (e.g. rivaroxaban, apixaban, phenprocoumon)
  - ☐ Injections for blood thinning (heparins)
  - ☐ others:________________________________
- ☐ no

Do you have or have you had any other known diseases?

- ☐ yes
  - ☐ Diabetes
  - ☐ Hypertension
  - ☐ Rheumatic disease
  - ☐ Tumor disease
  - ☐ others:________________________________
- ☐ no

part II

In your personal opinion, did the symptoms improve during treatment with IVIG infusions? If yes, after what period of time did you notice the first improvements?

- ☐ yes
  - ☐ 0 to 6 months
  - ☐ 6 months to 1 year
  - ☐ 1 year to 2 years
  - ☐ over 2 years
- ☐ no

Did treatment with IVIG infusions result in complete recovery of symptoms?

- ☐ yes
  - ☐ 0 to 6 months
  - ☐ 6 months to 1 year
  - ☐ 1 year to 2 years
  - ☐ over 2 years
- ☐ no

Has there been any recurrence or worsening of symptoms of the disease since treatment with IVIG infusions? If yes, after what period of time did you notice the worsening?

- ☐ yes
  - ☐ after 0 to 3 months
  - ☐ after 4 to 6 months
  - ☐ after 7 to 9 months
  - ☐ after 9 to 12 months
  - ☐ after 13 to 18 months
  - ☐ after more than 18 months
- ☐ no

Please evaluate the severity of your symptoms of the disease **before treatment** with IVIG infusions:

skin lesions	☐ intact skin<br>☐ erosions (top layer of skin affected)<br>☐ ulcers (deeper wound)
pain	☐ no pain<br>☐ moderate pain<br>☐ severe pain
restriction in everyday life	☐ none<br>☐ low<br>☐ severe<br>☐ very strong

Please evaluate the severity of your symptoms of the disease **at the present time**:

skin lesions	☐ intact skin<br>☐ erosions (top layer of skin affected)<br>☐ ulcers (deeper wound)
pain	☐ no pain<br>☐ moderate pain<br>☐ severe pain
restriction in everyday life	☐ none<br>☐ low<br>☐ severe<br>☐ very strong

part III

Did you experience any problems in your professional practice *due to your disease* at any time prior to receiving IVIG infusions at our clinic?

- ☐ yes
  - ☐ very strong problems
  - ☐ strong problems
  - ☐ minor problems
- ☐ no
- ☐ no answer possible, as not employed

Have you currently had any problems with IVIG infusions in our clinic in your professional practice *due to your disease*?

- ☐ yes
  - ☐ very strong problems
  - ☐ strong problems
  - ☐ minor problems
- ☐ no
- ☐ no answer possible, as not employed

Did you smoke before the onset of livedovasculopathy disease symptoms?

- ☐ yes
- ☐ no

Have you reduced smoking after receiving IVIG infusions at our clinic?

- ☐ yes, I have reduced the number of cigarettes per day
- ☐ yes, I have stopped smoking completely
- ☐ no, I did not reduce the number of cigarettes per day
- ☐ I have never smoked
